# Knowledge, Attitudes, and Practices Related to Antibiotic Use and Antibiotic Resistance Among Adults in Communities of Montserrado County, Liberia: A Household-Based Cross-Sectional Study

**DOI:** 10.3390/antibiotics15070680

**Published:** 2026-07-10

**Authors:** Bode Ireti Shobayo, Cecilia Stålsby Lundborg, Helena Nordenstedt, Tamba Fayiah, Mosoka Papa Fallah, Megha Sharma

**Affiliations:** 1 Department of Global Public Health, Karolinska Institutet, 17176 Stockholm, Sweden; cecilia.stalsby.lundborg@ki.se (C.S.L.); helena.nordenstedt@ki.se (H.N.); megha.sharma@ki.se (M.S.); 2 National Public Health Institute of Liberia, Oldest Congo Town Road, Monrovia P.O. Box 1871, Liberia; 3 Department of Medical Specialties, Danderyd University Hospital, 18288 Dandery, Sweden; 4 Department of Mathematics, University of Liberia, Capitol Hill, Monrovia P.O. Box 10-9020, Liberia; fayiaht@ul.edu.lr; 5 Africa Centers for Disease Control and Prevention, Addis Ababa P.O. Box 3243, Ethiopia; fallahm@africacdc.org; 6 Department of Pharmacology, Ruxmaniben Deepchand Gardi Medical College, Surasa, Ujjain 456006, India

**Keywords:** antibiotic use, antibiotic resistance (ABR), knowledge, attitude and practices (KAP), community-based survey, antibiotic stewardship, Liberia

## Abstract

**Background**: Inappropriate antibiotic use contributes significantly to antibiotic resistance (ABR), particularly in low- and middle-income countries. Understanding community-level knowledge, attitudes, and practices is essential for designing effective interventions. This study assessed Knowledge, Attitudes and Practices (KAP) related to antibiotic use, with consideration of awareness of antibiotic resistance, in Montserrado County, Liberia. **Methods**: A community-based cross-sectional survey was conducted among 1160 adults in Montserrado County, Liberia, using a structured questionnaire to assess knowledge, attitudes, and practices (KAP) related to antibiotic use and antibiotic resistance. Composite KAP scores were calculated from Likert-scale items and subsequently categorized into ordered levels (poor, moderate, and good) based on percentile thresholds. Ordinal logistic regression was used to examine associations between sociodemographic characteristics and KAP outcomes. Results are presented as crude and adjusted odds ratios (COR, AOR) with 95% confidence intervals (CI), and statistical significance was set at *p* < 0.05. **Results**: Participants demonstrated moderate knowledge, with correct identification of antibiotic use for bacterial infections (skin infections: 78.8%; ear infections: 76.8%), but widespread misconceptions were observed, including use for colds and flu (73.6%) and diarrhea (80.7%). Attitudes were mixed, with many recognizing inappropriate use, yet expectation-driven demand remained common (62.7% dissatisfied when antibiotics were not prescribed; 80.9% willing to suggest antibiotics to clinicians). Practices were inconsistent, with both appropriate and inappropriate behaviors reported. While 46.2% of participants always completed antibiotic courses, inappropriate practices were frequent, including antibiotic use for common symptoms (38.6%), preventive use (37.2%), and self-medication without prescription (20.1% always). In multivariable analysis, older age was associated with higher knowledge (AOR = 3.82, 95% CI: 1.26–11.1), while lower education predicted poorer knowledge and practices (e.g., AOR = 0.25, 95% CI: 0.07–1.00). Urban residence was associated with poorer practices (AOR = 0.42, 95% CI: 0.29–0.61). **Conclusions**: While overall knowledge of antibiotic use in Montserrado County is moderate, critical misconceptions remain, and appropriate practices are inconsistent. Weak relationships between knowledge, attitudes, and practices highlight the limitations of knowledge-based interventions alone. Efforts to improve antibiotic use should address structural drivers, including access to antibiotics, healthcare-seeking behavior, and regulatory enforcement, through targeted and context-specific strategies.

## 1. Introduction

Antibiotic resistance (ABR) arises when bacteria acquire the capability to survive exposure to antibiotics. It is a subset of antimicrobial resistance (AMR), and specifically refers to the ability of microorganisms such as bacteria, viruses, fungi, and parasites, to withstand drugs meant to kill them or inhibit their growth [1]. Despite various measures, ABR remains a growing global issue, emerging as one of the most significant public health threats today, where ABR occupies a major segment [2,3,4]. The widespread problem of ABR is largely attributed to the consequences of inappropriate use of antibiotics in various contexts, particularly in human healthcare, agriculture, and animal healthcare [5,6,7,8].

Irrational antibiotic use arises from a multifaceted set of factors, encompassing sociocultural and economic influences, deficiencies in education and training, inadequate regulatory policies, patient demand and self-medication, diagnostic uncertainty, prescriber and patient knowledge and behaviors, and ineffective communication between patients and providers [9,10,11]. Additional linkages for improper antibiotic usage are poverty, lack of access to healthcare services, and lack of community awareness [12,13,14]. A vast majority of antibiotic consumption is driven by the inadequate knowledge, attitude, and practices (KAP) regarding the use of antibiotics in the community [15,16].

A global survey spanning 76 countries reported a 65% increase in antibiotic consumption between 2000 and 2015, largely driven by substantial growth in low- and middle-income countries. Approximately 80% of antibiotics are used at the community level, with an estimated 20–50% of this use considered inappropriate [17]. The World Health Organization (WHO) subsequently recommended community involvement in combating ABR by a range of actions including minimizing the misuse of antibiotics, completing the entire course of treatment, refraining from sharing medicines with others, and not storing leftover antibiotics for subsequent use [18,19]. Additionally, the WHO introduced the Access, Watch, Reserve (AWaRe) classification, which categorizes antibiotics based on their resistance potential and guides appropriate use. Access antibiotics are recommended as first-line treatments, whereas Watch and Reserve antibiotics should be used more cautiously due to their higher risk of promoting resistance. This framework provides a standardized approach for monitoring antibiotic use and identifying patterns of misuse at the community level. Therefore, investigating the KAP considering antibiotic use at the community level is critical for developing policy recommendations and interventions. A number of recent systematic reviews have identified substantial gaps in knowledge regarding antibiotics, antibiotic resistance and antibiotic use, and the underlying drivers of resistance across low- and middle-income countries (LMICs) [16,20,21].

Community-based KAP studies conducted across LMICs consistently document pronounced and recurrent deficiencies in how antibiotics are understood and used at the population level. Surveys from sub-Saharan Africa including Ghana, Nigeria, Ethiopia, and Tanzania and from South and Southeast Asia, such as India, Bangladesh, and Nepal, report that between 40% and 80% of community members incorrectly believe antibiotics are effective for viral or self-limiting conditions, particularly colds, influenza, sore throat, and acute diarrhea [15,22,23,24]. These misconceptions are frequently accompanied by high levels of antibiotic self-medication, with reported prevalence ranging from approximately 30% to over 60% in many LMIC settings, largely driven by unrestricted over-the-counter access, weak enforcement of pharmaceutical regulations, and economic barriers that limit timely access to formal healthcare [15,21,24]. Importantly, several studies demonstrate that even among individuals with moderate or high levels of antibiotic awareness such as urban residents or those with secondary or tertiary education, inappropriate practices remain common, including premature discontinuation of treatment, prophylactic antibiotic use, and demand-driven acquisition without prescription [25,26]. This recurring pattern highlights a critical limitation of the traditional KAP model in LMIC contexts: knowledge does not reliably translate into appropriate attitudes or practices. The model assumes a linear progression from knowledge to action, which may not fully capture real-world decision-making. To better understand this gap, behavioral frameworks such as the Capability–Opportunity–Motivation–Behavior (COM-B) model suggest that behavior is shaped not only by knowledge (capability), but also by external factors such as access and regulation (opportunity), and internal drivers such as beliefs and expectations (motivation). This broader perspective is particularly relevant in contexts where structural and social factors may influence antibiotic use.

Recent systematic reviews and meta-analyses further emphasize that antibiotic misuse in community settings is strongly shaped by structural and contextual factors, including healthcare accessibility, medicine affordability, informal drug markets, and entrenched social norms favoring rapid symptom relief, rather than by knowledge deficits alone [27,28]. Collectively, this body of evidence underscores the need for context-specific, population-level studies that move beyond measuring awareness to examining how socioeconomic and health-system factors interact with individual beliefs to drive antibiotic-use behaviors which could impact ABR.

ABR is widely recognized as a major public health challenge across LMICs, yet representative data from low-income settings such as Liberia remain sparse [29]. In Liberia, systemic drivers, including limited access to quality healthcare, widespread availability of over-the-counter antibiotics, poor treatment adherence, and inadequate national surveillance, contribute to misuse and accelerate the emergence of resistant bacterial strains [30,31].

Despite these risks, empirical evidence on how antibiotics are understood and used at the community level is extremely limited. To date, no large-scale, household-based KAP studies have been published, constraining policymakers’ ability to design evidence-informed interventions or evaluate the effectiveness of the national AMR action plans [16,32,33]. This gap is particularly critical in Montserrado County, where high population density, concentration of pharmacies, and non-prescription antibiotic access create conditions conducive to misuse. Generating robust population-level KAP data is therefore essential to understanding community drivers of antibiotic use and resistance in Liberia, and informed the design of the present study. The objective of this study was to assess knowledge, attitudes, and practices related to antibiotic use, with consideration of awareness of antibiotic resistance among adults in Montserrado County, Liberia, examine the relationships between these domains, and determine sociodemographic factors associated with inappropriate practices to inform targeted antibiotic stewardship interventions.

## 2. Results

### 2.1. Sociodemographic Characteristics of Participants

A total of 1160 participants were included in the study. The majority of respondents were young adults, with 34% aged 18–29 years and 31% aged 30–39 years, while only 5.4% were aged ≥60 years. Most participants were male (62%), and over half had a junior high school education (51%), followed by 19% with a bachelor’s degree. A notable proportion (9.9%) had no formal education. In terms of occupation, the largest group was employed in the private sector (35%), followed by traders/self-employed (20%) and students (14%), while smaller proportions were farmers (10%) or government workers (8.9%). The majority of participants resided in urban areas (83%), and most did not have health insurance (94%). Socioeconomic status was generally low, with 63% reporting an annual household income below USD 1000. Access to healthcare services appeared relatively good, as 94% reported reaching a pharmacy within 30 min, and 77% could access a health facility within the same timeframe (Table 1).

### 2.2. Knowledge About Antibiotic Use and Awareness of Antibiotic Resistance

**Figure 1:**
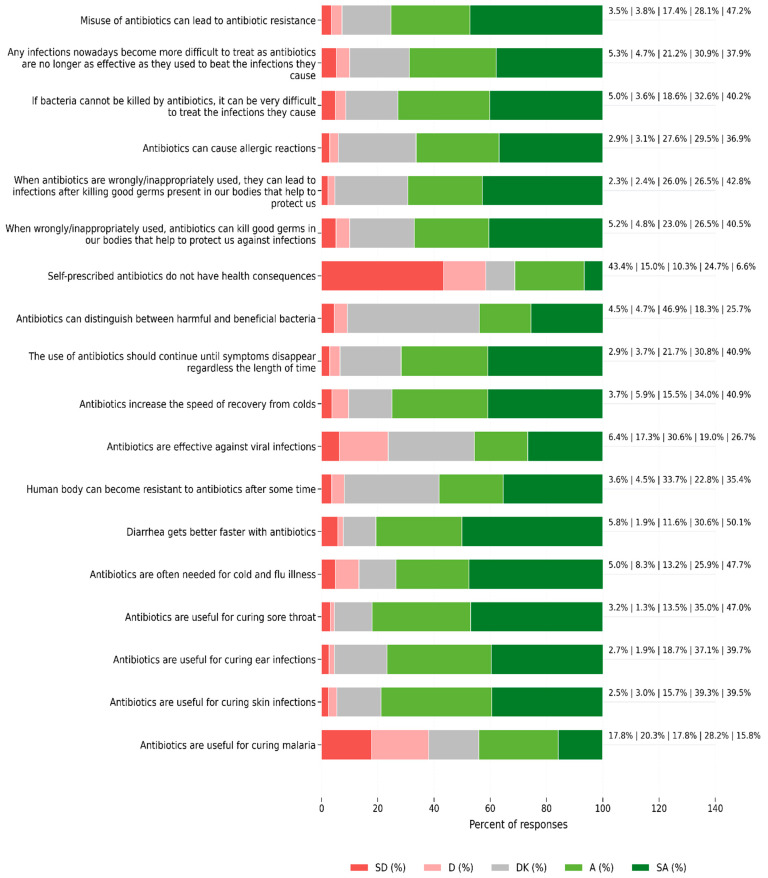
Participants’ responses to the questions related to knowledge about antibiotic use and awareness of antibiotic resistance in Liberia; SD = Strongly Disagree, D = Disagree, DK = Don’t Know, A = Agree, SA = Strongly Agree.

Participants demonstrated good knowledge of some aspects of antibiotic use. High proportions correctly identified that antibiotics are effective for bacterial infections such as skin infections (78.8%) and ear infections (76.8%). However, important misconceptions were widespread. A large proportion incorrectly believed that antibiotics are needed for colds and flu (73.6%) and that diarrhea resolves more quickly with antibiotics (80.7%). Knowledge regarding the ineffectiveness of antibiotics for viral infections was also limited. The lowest level of knowledge was observed regarding the consequences of self-prescription, where many respondents did not recognize associated health risks. However, this aggregate measure masks important variation across items, reflecting a pattern of moderate overall knowledge with substantial variability, where correct understanding of certain bacterial indications coexists with persistent misconceptions, particularly regarding viral illnesses such as colds and flu (Figure 1).

### 2.3. Attitudes Towards Antibiotic Use and Awareness of Antibiotic Resistance

**Figure 2:**
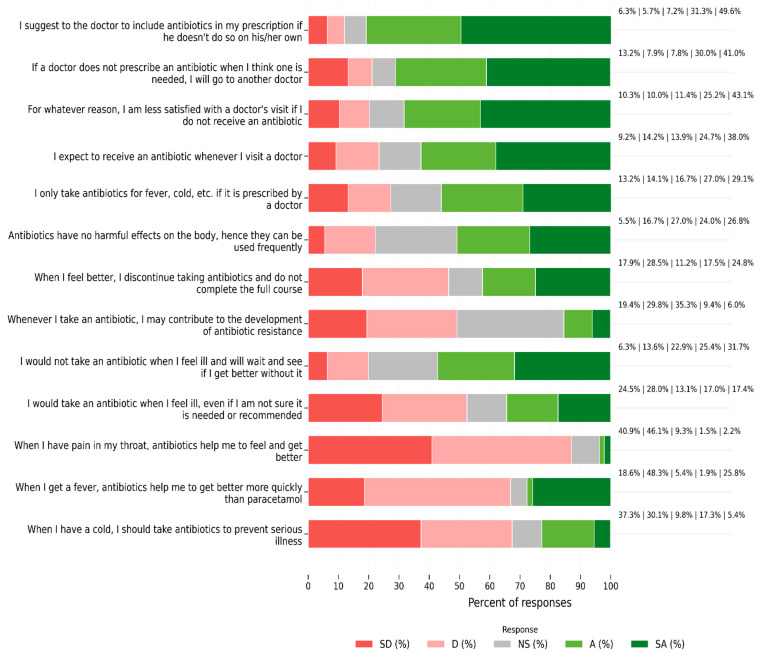
Responses to questions related to attitudes towards antibiotic use and awareness of antibiotic resistance; SD = Strongly Disagree, D = Disagree, NS = Not Sure, A = Agree, SA = Strongly Agree.

Responses to the attitude items revealed mixed perceptions. Most participants disagreed with inappropriate use of antibiotics for colds (67.4%) and throat pain (87.0%), indicating some awareness of appropriate use. However, uncertainty remained in decision-making, particularly regarding whether to take antibiotics when unsure of their necessity. Specifically, 52.5% of participants disagreed, while a substantial proportion (34.4%) expressed agreement and reflecting inconsistent attitudes toward appropriate antibiotic use. More than half of respondents (57.1%) reported willingness to delay antibiotic use and observe symptom progression. At the same time, expectations for antibiotic prescriptions were common. A majority (62.7%) reported dissatisfaction if antibiotics were not prescribed during medical visits and 80.9% indicated they would suggest antibiotics to a physician (Figure 2).

### 2.4. Practices Regarding Antibiotic Use and Awareness of Antibiotic Resistance

**Figure 3:**
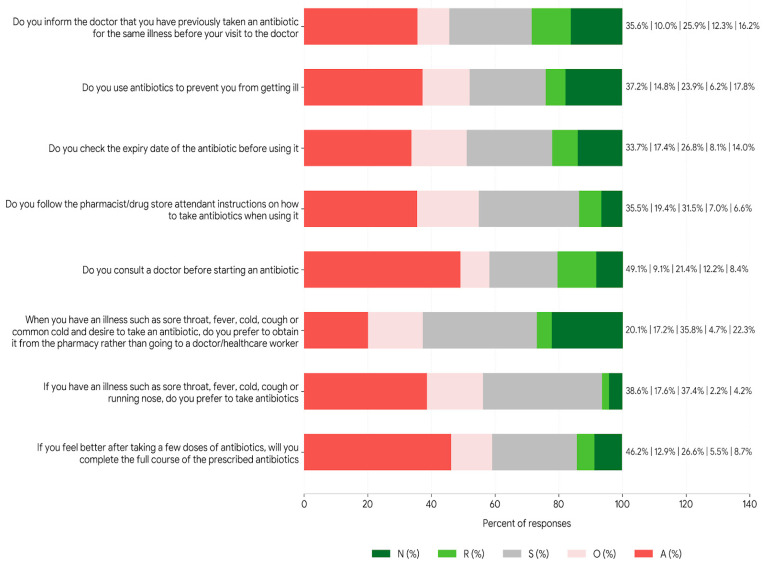
Responses to questions related to practices regarding antibiotic use and awareness of antibiotic resistance; N = Never, R = Rarely, S = Sometimes, O = Often, A = Always.

Self-reported practices showed considerable variation. Positive behaviors were observed in some areas: 46.2% of respondents reported always completing the full course of antibiotics, and 49.1% consistently consulted a doctor before use. However, inappropriate practices were also common. A substantial proportion reported frequent use of antibiotics for symptoms such as sore throat or fever (38.6% always), and 37.2% reported using antibiotics for preventive purposes. Additionally, 20.1% reported always obtaining antibiotics directly from pharmacies without consulting a doctor, while 35.8% did so occasionally. Other behaviors included inconsistent checking of expiry dates and incomplete communication with healthcare providers regarding prior antibiotic use (Figure 3).

In the bivariate analysis, age and education were significantly associated with knowledge of antibiotic use. Participants aged ≥ 60 years had significantly higher odds of better knowledge compared to those aged 18–29 years (COR = 5.16, 95% CI: 1.99–12.5, *p* < 0.001). Lower levels of education, particularly primary and junior high education, were significantly associated with poorer knowledge compared to those with bachelor’s degrees.

In the multivariable model, age ≥ 60 years remained a significant independent predictor of higher knowledge (AOR = 3.82, 95% CI: 1.26–11.1, *p* = 0.015). Education retained its significance, with respondents having primary education (AOR = 0.25, 95% CI: 0.07–1.00, *p* = 0.043) and vocational education (AOR = 0.10, 95% CI: 0.02–1.04, *p* = 0.029) demonstrating significantly lower odds of good knowledge compared to those with a bachelor’s degree. Other variables, including gender, residence, and occupation, were not significantly associated with knowledge after adjustment (Table 2).

Given the clustered sampling design, standard errors may be underestimated, and therefore *p*-values, particularly those close to the threshold for statistical significance, should be interpreted cautiously.

In the bivariate analysis, residence and occupation were significantly associated with attitudes toward antibiotic use. Urban residents had higher odds of positive attitudes compared to rural residents (COR = 1.46, 95% CI: 1.03–2.08, *p* = 0.032). Participants working in the private sector (COR = 1.98, 95% CI: 1.23–3.16, *p* = 0.005) and those who were traders/self-employed (COR = 1.67, 95% CI: 1.01–2.78, *p* = 0.047) also showed significantly more positive attitudes compared to farmers.

In the multivariable analysis, only occupation in the private sector remained a statistically significant predictor of positive attitudes (AOR = 1.86, 95% CI: 1.05–3.28, *p* = 0.034). The association with urban residence and other occupational categories was no longer significant after adjustment. Age, gender, and education were not significantly associated with attitudes in both bivariate and multivariable models (Table 3).

In the bivariate analysis, several sociodemographic factors, including residence, occupation, and education, were significantly associated with antibiotic use practices. Urban residence was associated with significantly lower odds of better practice compared to rural residence (COR = 0.38, 95% CI: 0.28–0.52, *p* < 0.001). Similarly, individuals working in the government, private sector, trade/self-employment, and those who were unemployed had significantly lower odds of appropriate practices compared to farmers. Lower levels of education, particularly junior high and no formal education, were also significantly associated with poorer practices in the unadjusted model.

However, after adjusting for confounders, most of these associations lost statistical significance. Urban residence remained a strong independent predictor, with significantly lower odds of good practice (AOR = 0.42, 95% CI: 0.29–0.61, *p* < 0.001). Education at the junior high level remained significantly associated with lower odds of appropriate practices (AOR = 0.67, 95% CI: 0.47–0.96, *p* = 0.029), while other education categories were not statistically significant in the adjusted model. No significant associations were observed for age or gender (Table 4).

## 3. Discussion

To our knowledge, this study represents the first representative household survey to examine KAP regarding antibiotic use and awareness of antibiotic resistance in Liberia, providing a comprehensive assessment among 1160 adults in Montserrado County. The findings demonstrate a complex pattern, characterized by moderate levels of knowledge, mixed attitudes, and inconsistent practices, with substantial variability across individuals. Importantly, the results reveal that knowledge and attitudes do not consistently translate into appropriate practices, highlighting a disconnect between awareness and behavior.

Overall, participants demonstrated moderate knowledge, with correct identification of antibiotic effectiveness for bacterial infections such as skin infections (78.8%) and ear infections (76.8%). However, this was offset by widespread misconceptions, particularly regarding inappropriate indications. For example, a large proportion of respondents incorrectly believed that antibiotics are needed for colds and flu (73.6%) and that diarrhea resolves more quickly with antibiotics (80.7%). The widespread belief in antibiotics for viral syndromes is not unique to Liberia as similar rates have been reported in studies across sub-Saharan Africa and Asia, indicating a persistent global public health challenge [34,35,36,37]. These findings are also consistent with community-based studies in India and other low- and middle-income countries (LMICs), where between 40% and 80% of respondents incorrectly associate antibiotics with viral illnesses. This pattern reflects a fragmented understanding of antibiotic function, where correct knowledge of specific bacterial indications coexists with fundamental misconceptions [38]. Importantly, knowledge regarding the risks associated with inappropriate use, including self-prescription, was relatively low, representing one of the weakest areas of understanding. This finding is notably concerning, as self-medication is a recognized contributor to ABR in LMICs, where antibiotics are often accessible without a prescription [39,40]. This knowledge gap, coupled with the high proportion of participants (35.8%) who “sometimes” obtain antibiotics directly from a pharmacy, creates a fertile ground for the proliferation of ABR. The lack of understanding about the personal and societal health consequences of self-prescription represents a critical target for future educational interventions.

Participants’ attitudes toward antibiotic use were moderate but internally inconsistent. While a majority rejected inappropriate antibiotic use for certain conditions, such as colds (67.4%) and throat pain (87.0%), a substantial proportion reported demand-driven expectations for antibiotics. Specifically, 62.7% reported dissatisfaction if antibiotics were not prescribed and 80.9% indicated they would suggest antibiotics to a doctor. These findings highlight a critical contradiction: awareness does not prevent expectation-driven behavior. Even when participants understand appropriate use, they may still seek antibiotics due to perceived benefits or prior experiences. Similar patterns have been reported in studies from Ghana, Nigeria, and India, where patient expectations strongly influence prescribing behavior [38,41]. This suggests that antibiotic misuse is not solely a knowledge issue but also reflects behavioral norms and healthcare system dynamics. This highlights the need for targeted behavioral change interventions that go beyond awareness to address expectations around clinical care, strengthen patient–provider communication, and support prescribers in adhering to rational antibiotic use guidelines despite external pressure.

The mixed attitudes on the decision to delay antibiotic treatment for an illness imply that a portion of the population is amenable to more prudent usage. Nonetheless, this is eclipsed by the pronounced tendency to pursue and expect antibiotics. These findings are especially significant because they echo, but also meaningfully diverge from, household and community surveys conducted in other low- and middle-income countries. For example, a large community-based study in Ghana and Burkina Faso found that although most adults had heard of antibiotics, only 34.2% in Ghana and 26.7% in Burkina Faso were aware of antimicrobial resistance, and many reported antibiotic practices that did not align with their stated understanding, highlighting a disconnect between awareness and behavior in general populations [42]. When compared to these settings, our findings underscore the critical importance of addressing community-level drivers of antibiotic misuse in Liberia. They show that Montserrado communities face challenges similar to those seen internationally, but also reveal context-specific gaps that require targeted interventions.

Practices represented the most concerning domain, with substantial evidence of inappropriate antibiotic use despite moderate knowledge levels. The positive practices, such as a high rate of completing the full antibiotic course (46.2% always) and consulting a doctor (49.1% always), are encouraging and represent behavioral cornerstones of responsible antibiotic use. However, these are counterbalanced by high rates of inappropriate practices, including the use of antibiotics for prevention (37.2% always) and a preference for taking antibiotics for common viral symptoms like sore throat and fever (38.6% always). The practice of preventive antibiotic use is particularly concerning, as it reflects a deep-seated misconception and contributes significantly to unnecessary antibiotic exposure and resistance [43]. Encouraging behaviors, such as completing the full antibiotic course (46.2% always) and consulting a doctor before use (49.1% always), contrast with broader evidence showing that inappropriate antibiotic practices are widespread in many general-population settings. A scoping review of KAP studies across vulnerable communities globally found that self-medication was pervasive and appropriate antibiotic use was consistently poor, with little consideration for when antibiotics are truly needed [44]. Similarly, a community survey among adults in Shaqra, Saudi Arabia, showed only 60.9% demonstrating good antibiotic practices, with significant gaps in appropriate use despite relatively high knowledge and attitude scores, indicating that large segments of the general population still engage in misuse [45]. When viewed against these studies, the comparatively stronger adherence to key responsible behaviors found in our study becomes even more notable. Still, the persistent practices in our study, such as frequent preventive antibiotic use (37.2%) and the use of antibiotics for viral symptoms like sore throat and fever (38.6%), remain concerning and highlight areas needing urgent intervention. The coexistence of moderate knowledge and inappropriate practices strongly supports the concept of the “KAP gap” [46], where awareness does not lead to behavior change. Similar findings were reported in a large community study in Delhi, India, where substantial antibiotic misuse persisted despite moderate knowledge levels [26].

The regression analysis in our study revealed that sociodemographic characteristics significantly influenced KAP outcomes, particularly knowledge and practices. Education emerged as a key determinant, with individuals with lower levels of education having reduced odds of being in higher knowledge and practice categories. This association should not be interpreted as a direct causal relationship, but rather as reflecting the influence of broader contextual and structural factors. For example, individuals with lower levels of formal education, particularly those in rural or agricultural settings, may have distinct patterns of healthcare access, greater reliance on traditional practices, and less exposure to over-the-counter antibiotic markets, all of which may shape reported practices. Importantly, this pattern is consistent with emerging evidence from LMICs including Liberia showing that higher knowledge or education does not necessarily translate into appropriate antibiotic-use practices [47,48]. Instead, it underscores the critical role of opportunity, access, and health system context in shaping behavior, beyond knowledge alone. Occupation and residence also played important roles. Urban residence and certain occupational groups were associated with differences in antibiotic-use practices, likely reflecting variations in access, affordability, and exposure to healthcare systems. These findings align with growing evidence that antibiotic-use behavior is shaped by contextual and structural factors, rather than knowledge alone [49,50]. For example, easier access to pharmacies in urban areas may increase opportunities for self-medication, while economic constraints may influence how antibiotics are used or prioritized.

HCWs in both government and private sectors did not demonstrate significantly better knowledge or practices compared with other occupational groups in this study, with no statistically significant associations observed after adjustment. Although government HCWs showed a tendency toward more positive attitudes in unadjusted analysis, this was not sustained in multivariable models, suggesting that professional status alone does not ensure appropriate antibiotic-use behavior. This pattern aligns with findings from other LMIC settings, including studies in sub-Saharan Africa, where HCWs may possess adequate knowledge but still engage in inappropriate practices due to systemic constraints. such as diagnostic uncertainty, patient demand, and weak regulatory environments [51]. However, the absence of a clear advantage among HCWs in this study highlights important potential implications, including possible gaps in continuing professional education, inconsistent exposure to antibiotic stewardship principles, and the influence of health system constraints such as diagnostic limitations and patient pressure. Together, these findings suggest that HCWs are influenced by the same structural and behavioral drivers as the general population, underscoring the need for system-wide stewardship interventions that strengthen both provider capacity and enabling environments.

A central finding of this study is the lack of alignment between knowledge, attitudes, and practices. Despite moderate knowledge, inappropriate practices remained common, indicating that the traditional linear KAP model does not adequately explain behavior in this setting. This finding is consistent with global evidence demonstrating that antibiotic-use behavior is influenced by the interplay of knowledge (capability), access and environment (opportunity), and beliefs and expectations (motivation). These findings can be further understood through established behavioral frameworks such as the COM-B model which was introduced earlier. According to this model, behavior is not determined by knowledge alone (capability), but also by opportunity (e.g., access to antibiotics, healthcare systems, and regulatory environment) and motivation (e.g., beliefs, expectations, and social norms). In this study, although participants demonstrated moderate knowledge and awareness, inappropriate practices persisted, suggesting that knowledge (capability) was insufficient to drive behavior change in the absence of supportive structural conditions and aligned motivations. The widespread availability of antibiotics without prescription, combined with patient expectations and healthcare access constraints, likely represents key opportunity and motivational drivers of misuse. This interpretation highlights the limitations of the traditional linear KAP model and supports a more comprehensive, systems-based approach to behavior change.

These findings should also be interpreted within the broader geographic and health-system context of the study setting. Montserrado County is more highly urbanized than most other regions of Liberia, and this likely influences patterns of antibiotic use through greater access to healthcare facilities, pharmacies, and over-the-counter medicines. Accordingly, caution is warranted in extrapolating these findings to more rural areas of the country, where access, availability, and health-seeking behaviors may differ substantially. However, as the most populous and economically active region of Liberia, Montserrado provides critical insight into antibiotic-use behaviors in contexts where access to antibiotics is relatively high and misuse is most likely to occur. Additionally, given the cross-sectional design of the study, the observed relationships should be interpreted as associations rather than causal effects.

The findings of this study have important implications for antibiotic stewardship and public health policy in Liberia. The absence of a significant relationship between knowledge, attitudes, and practices indicates that interventions focused solely on improving awareness are unlikely to produce meaningful behavioral change. Instead, strategies should prioritize addressing the structural and contextual drivers of antibiotic use identified in this study. These include strengthening regulation and enforcement to limit non-prescription antibiotic sales, improving access to affordable and timely healthcare to reduce self-medication, and supporting healthcare providers in managing patient expectations for antibiotics.

In addition, the strong influence of sociodemographic factors, particularly occupation, education and residence highlights the need for targeted and segmented interventions. Instead of relying solely on awareness campaigns, interventions should prioritize strengthening enforcement of regulations on antibiotic sales, reducing over-the-counter access, improving access to affordable and timely healthcare, and addressing patient expectations that drive demand-based prescribing. Given the observed sociodemographic differences, targeted approaches are essential. For instance, education campaigns tailored to lower-literacy populations can improve understanding of appropriate antibiotic use, while behavior-focused interventions in higher-access urban settings can address misuse linked to convenience and demand.

Taken together, these findings underscore that effective antibiotic stewardship in Liberia requires a multifaceted approach combining regulatory, health-system, and behavior-change strategies, rather than relying on knowledge-based interventions alone.

Future research is needed to further understand the drivers of antibiotic-use behavior in this context. In particular, qualitative studies could provide deeper insight into the social, cultural, and behavioral factors influencing antibiotic use, including patient expectations, perceptions of illness, and decision-making processes. Longitudinal studies would also be valuable for assessing changes in knowledge, attitudes, and practices over time and for evaluating the impact of interventions. In addition, research examining healthcare provider behaviors, prescribing practices, and regulatory environments is essential to better understand the system-level factors contributing to antibiotic misuse. Finally, intervention-based studies designed to address structural barriers such as access to healthcare and over-the-counter availability of antibiotics are needed to identify effective strategies for improving antibiotic stewardship in similar settings.

## 4. Methods

### 4.1. Study Setting and Population

A community-based cross-sectional study was conducted to assess knowledge, attitudes, and practices related to antibiotic use and awareness of antibiotic resistance in Montserrado County from September to December 2024. As per Liberia Population and Housing Census 2022, Montserrado County is the most populous county in the country, with a population of 1.9 million, accounting for about one quarter of the total population of Liberia [52]. It is Liberia’s only urban county and hosts the capital city of Liberia, Monrovia. The county is divided into seven health districts, i.e., four urban (Central Monrovia, Bushrod, Commonwealth, and Somalia Drive) and three rural (Careysburg, St. Paul, and Todee). Most of the healthcare facilities and diagnostic services, such as hospitals, laboratories, and pharmacies, are concentrated in the county. Many registered and well-organized pharmacies, along with informal medicine shops, serve the people of Montserrado County, including selling antibiotics, over-the-counter, without prescription. This practice, which is also seen in other parts of the country, allows for unrestricted access to antibiotics.

### 4.2. Sample Size and Study Design

The minimum sample size required for the study was estimated at 385 participants using the Raosoft sample size calculator, assuming a 95% confidence level, 5% margin of error, and a response distribution of 50%, which provides the most conservative estimate for population-based surveys. To enhance the precision of estimates and allow for more reliable subgroup analyses across sociodemographic categories, a larger target sample size of 1200 participants was planned. Given the use of a multistage cluster sampling design, the sample size was adjusted to account for the design effect (Deff), which reflects increased variance due to clustering of responses within communities. A design effect of 2.0 was assumed, consistent with similar community-based surveys. Accordingly, the minimum sample size (*n* = 385) was adjusted to 770 (385 × 2.0). To enable reliable subgroup analyses and improve estimate stability across sociodemographic strata, the final target sample size was further increased to 1200 participants. With 10 clusters included in the study, this corresponds to an average cluster size of approximately 120 participants per cluster. This approach is consistent with recommendations for community-based KAP studies, where increased sample size improves estimate stability and reduces sampling variability rather than being solely intended to increase statistical power [53,54]. The sampling frame consisted of all communities within the seven health districts of Montserrado County. Given the relatively uniform cluster sizes and the descriptive focus of the analysis, unweighted estimates were considered appropriate. The study included adult residents (≥18 years) residing in Montserrado County who were present in their homes at the time of data collection and were willing to participate and provide written informed consent. Montserrado County is predominantly urban and includes the capital city, Monrovia. Therefore, the higher proportion of urban participants in the sample reflects the underlying demographic distribution of the study area rather than sampling imbalance.

Ten communities were randomly chosen from the seven health districts based on sample probabilities proportionate to population size to ensure that larger communities had a higher probability of selection. A community was defined as “a group of people living in the same geographic location (such as a town, neighborhood, or district) who share common social, cultural, economic, or environmental characteristics, and often rely on the same health, education, or administrative services” [55]. The total number of houses in each community was obtained from the records of the community head. In each selected community, the residence of the community leader was used solely as a neutral geographic starting point. The direction of household selection from this point was determined by randomly selecting a direction by spinning a pen. From the selected direction, households were approached sequentially using a fixed sampling interval (*n* = N/10), ensuring that all households in the sampling frame had an approximately equal probability of selection regardless of proximity to the starting point. This approach minimizes selection bias associated with convenience or purposive entry points. Within each household, the head of household was initially approached due to their availability and familiarity with household health decisions. However, to reduce gender and power-related selection bias, any eligible adult (≥18 years) present at the time of the visit was invited to participate if the head was unavailable or declined. This flexible respondent selection approach increased inclusivity and ensured broader representation of perspectives within households. Given the multistage sampling design, participants were nested within communities; however, analyses were conducted at the individual level.

A structured questionnaire was used to assess knowledge, attitudes, and practices related to antibiotic use and awareness of antibiotic resistance. The instrument was adapted from a previously validated KAP tool used in a similar community-based study [56], with modifications to reflect the local Liberian context, through refinement of wording, inclusion of contextually relevant examples, and adjustment of item phrasing to improve comprehension (Supplementary File S1: Study Questionnaire). Adaptations included refinement of wording for clarity, contextual relevance of examples, and minor adjustments to response framing to ensure cultural appropriateness. The questionnaire which was in English comprised four sections: information on socio-demography, KAP relating to antibiotic use and awareness of antibiotic resistance. Data were collected through face-to-face interviews conducted by trained data collectors. To ensure comprehension among participants with varying levels of education, including those with no formal schooling, questions were verbally translated into Liberian colloquial English during the interview process.

Prior to data collection, the instrument was pilot-tested among a small sample of adults (*n* = 10) in a community not included in the final study sample to assess clarity, comprehension, and flow of questions. Feedback from the pilot testing was used to revise ambiguous items, improve wording, and ensure that questions were easily understood by respondents. Following pilot testing, the internal consistency of the final questionnaire was assessed using Cronbach’s alpha, yielding values of 0.77 for knowledge, 0.70 for attitudes, and 0.64 for practices, indicating acceptable internal consistency for exploratory research, although the reliability of the practice domain was comparatively lower than that of knowledge and attitudes. Lower internal consistency in the practice domain may reflect the heterogeneous nature of self-reported behaviors, which are often linked to varying contextual and situational factors. While the Cronbach’s alpha value (α = 0.64) is slightly below the conventional threshold of 0.70, it remains acceptable for exploratory research, particularly in behavioral studies where constructs are inherently diverse. However, the lower internal consistency of the practice domain may introduce some measurement variability and should be considered when interpreting practice-related findings. Content validity was ensured through review by subject-matter experts prior to field implementation.

Demographic data comprised information on age, sex, educational status, area of residence (urban/rural), health insurance, occupation, type of family (nuclear/extended) and average annual income of family (cost estimates were recorded in Liberian Dollars (LRD), with conversions to United States Dollars (USD), reflecting Liberia’s dual-currency economy). The primary outcome was antibiotic-use practice, while knowledge and attitude scores were treated as secondary outcomes. To minimize social desirability bias, interviews were conducted anonymously, and no personal identifiers were recorded. Data collectors were trained to maintain neutrality, avoid leading questions, and emphasize that there were no right or wrong answers. Respondents were assured that their responses would remain confidential.

### 4.3. Inclusion and Exclusion Criteria

Participants were eligible for inclusion if they were adults aged ≥18 years, residing in the selected households in Montserrado County, present at the time of data collection, and willing to provide informed consent.

Individuals were excluded if they were unwilling to participate or were incapable of providing informed consent or completing the interview. Incapacity in this context included individuals with cognitive impairment, severe illness, or communication barriers (e.g., language difficulties or inability to comprehend or respond to interview questions) that prevented effective participation.

### 4.4. Measurement and Operationalization of KAP

Knowledge, attitudes, and practices related to antibiotic use and awareness of antibiotic resistance were assessed using a structured questionnaire comprising 18 knowledge items, 13 attitude items, and 8 practice items. All items were measured on five-point Likert-type scales. Knowledge and attitude items were scored from 1 (strongly disagree) to 5 (strongly agree), while practice items were scored from 1 (never) to 5 (always). To ensure consistency in interpretation, items for which agreement or higher frequency reflected incorrect knowledge, inappropriate attitudes, or undesirable practices were reverse-coded prior to analysis. Following reverse coding, higher scores across all three domains consistently indicated better knowledge, more appropriate attitudes, and more appropriate antibiotic-use practices.

For each respondent, composite scores for knowledge, attitudes, and practices were calculated by averaging item responses within each domain. This approach generated continuous scores ranging from 1 to 5. The knowledge score represented the respondent’s overall understanding of appropriate antibiotic use and awareness of antibiotic resistance. The attitude score reflected beliefs, perceptions, and expectations regarding antibiotic use, including patient–provider interactions. The practice score represented self-reported behaviors related to antibiotic use, such as adherence, healthcare-seeking, and acquisition of antibiotics. The primary outcome of interest in this study was the practice score, as it most directly reflects behaviors contributing to antibiotic misuse and ABR. Knowledge and attitude scores were treated as secondary outcomes, representing upstream determinants of behavior.

For analytical purposes, composite scores for knowledge, attitudes, and practices were categorized into three ordered levels (poor, moderate, and good) for each domain. Categorization was based on percentile thresholds—where scores at or below the 25th percentile were classified as poor, those between the 25th and 75th percentile as moderate, and those above the 75th percentile as good—and intended as descriptive groupings rather than standardized or validated classifications. This approach ensured a balanced distribution of participants across categories and allowed for meaningful interpretation of ordinal outcomes. These ordered categories were subsequently used as dependent variables in regression analyses, with higher categories representing better knowledge, more positive attitudes, and more appropriate antibiotic-use practices. The composite practice score, while useful for summarizing behavior, may mask variability across individual behaviors and should be interpreted with caution.

### 4.5. Data Analysis

Data were entered, cleaned, and verified for completeness in Microsoft Excel 2016, then exported to R version 4.6.0 for analysis. Descriptive statistics summarized sociodemographic characteristics and KAP variables. Categorical variables were presented as frequencies and percentages. Associations between sociodemographic factors and categorized knowledge, attitude, and practice outcomes were assessed using ordinal logistic regression (proportional odds model). The categorized knowledge, attitude, and practice variables (poor, moderate, and good) were used as dependent variables in the ordinal regression models. Univariable models estimated crude odds ratios (COR), followed by multivariable models to obtain adjusted odds ratios (AOR) with 95% confidence intervals. Statistical significance was set at *p* < 0.05. Sampling weights were not applied in the analysis, which may affect the representativeness of estimates across communities of differing population sizes.

### 4.6. Ethical Considerations

The study was conducted after obtaining ethical approval from the National Research Ethics Board of Liberia (NREB-009-22). Data collection was started only after obtaining written consent from the participant. Data were recorded anonymously, and confidentiality was ensured by storing data under restricted access in password-protected computers. 

## 5. Strengths and Limitations

This study represents the first large, community-based household survey assessing antibiotic-related knowledge, attitudes, and practices (KAP) in Montserrado County, Liberia, addressing a critical evidence gap in a setting where data on drivers of antibiotic resistance are limited. A rigorous multistage sampling strategy was employed across both urban and rural districts, yielding a representative cross-section of the county’s diverse population. The study is further strengthened by its large sample size, high response rate (97%), and use of a validated, contextually adapted KAP instrument.

The use of ordinal logistic regression to model categorized KAP outcomes represents a methodological strength, as it appropriately accounts for the ordered nature of the data and allows for more robust interpretation of associations compared to traditional continuous or binary approaches.

Several limitations should be considered when interpreting the findings. First, the cross-sectional design precludes causal inference, and observed relationships should be interpreted as associations. Second, although a multistage sampling approach was used, the initial selection of households from community leaders’ residences may have introduced minor spatial clustering; however, systematic interval sampling was applied to minimize this risk. Given the multistage sampling design, participants were clustered within communities; however, clustering was not explicitly accounted for in the analysis. This may have led to underestimation of standard errors and overstatement of statistical significance. While the sample was relatively large and distributed across multiple communities, this does not fully address the potential impact of clustering. Future analyses using multilevel or cluster-adjusted models are warranted. Also, sampling weights were not applied in the analysis, which may affect the representativeness of estimates across communities of different population sizes.

Potential biases related to data collection should also be acknowledged. As with most KAP studies, all measures were self-reported and therefore subject to social desirability bias, which may have led to over-reporting of appropriate antibiotic-use practices and under-reporting of inappropriate behaviors. However, efforts were made to reduce these through anonymous data collection, neutral interviewing techniques, and standardized procedures. The initial approach to household heads may also have introduced some intra-household selection bias.

From an analytical perspective, although key sociodemographic variables were included, important confounders such as detailed income measures, healthcare access, and broader health system factors were not directly measured or included in the analysis, which may limit interpretation and contribute to residual confounding. Additionally, the internal consistency of the practice domain was slightly below the conventional threshold (Cronbach’s α = 0.64), indicating moderate reliability. This likely reflects the heterogeneous and context-dependent nature of self-reported behaviors, which may introduce measurement variability and should be considered when interpreting practice-related findings.

Finally, although the study was conducted in Montserrado County—the most populous and urbanized region of Liberia—findings may not be fully generalizable to more rural areas with different healthcare access and sociocultural contexts. Despite these limitations, the study provides robust and valuable insights into community-level antibiotic use and awareness of antibiotic resistance within the study setting.

## 6. Conclusions

This study demonstrates that antibiotic-use behavior in Montserrado County is shaped by a complex interaction of knowledge, attitudes, and contextual factors, with moderate knowledge coexisting alongside persistent misconceptions and widespread inappropriate practices. Importantly, the lack of alignment between knowledge and behavior highlights the limitations of awareness-based approaches alone. Antibiotic-use practices were significantly associated with sociodemographic characteristics, reflecting the critical role of structural factors such as access to healthcare, availability of antibiotics, and socioeconomic conditions.

These findings indicate that improving antibiotic use in this setting requires multi-level interventions that extend beyond education to address the broader health system and regulatory environment. Strengthening enforcement of antibiotic dispensing policies, improving access to affordable healthcare, and designing targeted, context-specific behavior change strategies will be essential for reducing misuse and mitigating the growing threat of antibiotic resistance.

From a policy and practice perspective, several targeted actions are warranted; however these recommendations should be interpreted with caution, as this study did not evaluate the effectiveness of specific interventions. First, regulatory authorities should strengthen enforcement of existing policies restricting over-the-counter antibiotic sales, particularly in community pharmacies and informal drug outlets. Second, healthcare providers should be supported through training and clinical guidelines to manage patient expectations and reduce demand-driven prescribing. Third, community-based interventions should be tailored to specific population groups: for example, communication strategies targeting higher-income or more educated populations should address unnecessary demand for antibiotics, while interventions for lower-resource communities should emphasize appropriate use and adherence. Finally, improving access to affordable and timely healthcare services may reduce reliance on self-medication and promote appropriate antibiotic use. Collectively, these strategies highlight that reducing inappropriate antibiotic practices requires more than knowledge; it requires transforming the social, economic, and structural conditions that shape everyday health decisions. These context-specific and multi-level strategies are critical for informing the implementation of Liberia’s National Action Plan on antimicrobial resistance.

## Figures and Tables

**Table 1:** Descriptive characteristics of study participants in Montserrado, Liberia (N = 1160).

Characteristic	N = 1160 ^1^
**Age group (years)**	
18–29	392 (34%)
30–39	356 (31%)
40–49	224 (19%)
50–59	125 (11%)
60+	63 (5.4%)
**Gender**	
Male	723 (62%)
Female	437 (38%)
**Education**	
No formal Edu	115 (9.9%)
Primary	42 (3.6%)
Junior High	594 (51%)
High School	108 (9.3%)
Bachelor	215 (19%)
Masters	75 (6.5%)
Vocational	11 (0.9%)
**Occupation**	
Farmer/Agriculture	120 (10%)
Government worker	103 (8.9%)
HCW-Government	29 (2.5%)
Private Sector	407 (35%)
HCW-private	13 (1.1%)
Student	157 (14%)
Trader/Self-employed	235 (20%)
Unemployed	96 (8.3%)
Retired	0 (0%)
**Have health Insurance**	
No	1093 (94%)
Yes	67 (5.8%)
**Residence**	
Rural	202 (17%)
Urban	958 (83%)
**Avg income of family/year (USD)**	
<1000	739 (64%)
1000–3999	391 (33%)
4000–6999	24 (2.1%)
7000–9999	4 (0.3%)
10,000+	2 (0.2%)
**Time to reach nearest pharmacy (minutes)**	
<30	1086 (94%)
30–60	72 (6.2%)
61–120	2 (0.2%)
**Time to reach nearest HF (minutes)**	
<30	897 (77%)
30–60	253 (22%)
61–120	10 (0.9%)

^1^ *n* (%); Avg—Average; HF—Health Facility, HCW—Healthcare worker.

**Table 2:** Ordinal regression of antibiotic use knowledge and sociodemographic factors, Montserrado, Liberia, 2025 (N = 1160).

	Bivariate			Multivariate		
Characteristic	Crude OR	95% CI	*p*-Value	Adjusted OR	95% CI	*p*-Value
**Age group (years)**						
18–29	—	—		—	—	
30–39	1.11	0.58, 2.13	0.748	0.93	0.46, 1.90	0.837
40–49	1.15	0.55, 2.40	0.714	0.92	0.40, 2.10	0.844
50–59	1.92	0.79, 4.51	0.142	1.58	0.60, 4.12	0.352
60+	5.16	1.99, 12.5	**<0.001**	3.82	1.26, 11.1	**0.015**
**Gender**						
Male	—	—		—	—	
Female	0.90	0.54, 1.51	0.694	0.85	0.47, 1.50	0.565
**Residence**						
Rural	—	—		—	—	
Urban	0.54	0.29, 1.02	0.055	0.53	0.26, 1.10	0.083
**Occupation**						
Farmer/Agriculture	—	—		—	—	
Government worker	0.88	0.28, 2.75	0.827	1.64	0.47, 5.69	0.436
HCW-Government	1.35	0.24, 6.32	0.724	1.75	0.28, 9.66	0.543
Private Sector	0.81	0.34, 1.97	0.632	1.50	0.54, 4.19	0.442
HCW-private	0.73	0.08, 6.90	0.807	1.79	0.17, 17.8	0.649
Student	0.58	0.21, 1.64	0.300	1.07	0.32, 3.57	0.907
Trader/Self-employed	1.01	0.40, 2.62	0.991	1.58	0.56, 4.49	0.393
Unemployed	1.52	0.50, 4.58	0.457	1.82	0.54, 6.03	0.328
**Education**						
Bachelor	—	—		—	—	
Primary	0.20	0.06, 0.82	**0.019**	0.25	0.07, 1.00	**0.043**
High School	0.66	0.24, 1.73	0.414	0.87	0.31, 2.40	0.799
Junior High	0.46	0.24, 0.91	**0.025**	0.74	0.34, 1.60	0.442
Masters	0.78	0.25, 2.24	0.654	1.05	0.31, 3.31	0.934
No formal Edu	0.65	0.24, 1.67	0.381	0.92	0.32, 2.63	0.882
Vocational	0.12	0.02, 1.28	**0.039**	0.10	0.02, 1.04	**0.029**

Abbreviations: CI = Confidence Interval, OR = Odds Ratio, Significant *p*-values (*p* < 0.05) are shown in bold.

**Table 3:** Ordinal regression of antibiotic use attitudes and sociodemographic factors, Montserrado, Liberia, 2025 (N = 1160).

	Bivariate			Multivariate		
Characteristic	Crude OR	95% CI	*p*-Value	Adjusted OR	95% CI	*p*-Value
**Age group (years)**						
18–29	—	—		—	—	
30–39	1.06	0.76, 1.47	0.738	1.07	0.74, 1.53	0.720
40–49	0.90	0.62, 1.32	0.605	0.96	0.63, 1.47	0.860
50–59	0.90	0.57, 1.43	0.654	1.06	0.63, 1.78	0.819
60+	1.01	0.54, 1.87	0.973	1.21	0.60, 2.40	0.592
**Gender**						
Male	—	—		—	—	
Female	0.93	0.71, 1.23	0.620	0.89	0.66, 1.21	0.457
**Residence**						
Rural	—	—		—	—	
Urban	1.46	1.03, 2.08	**0.032**	1.22	0.80, 1.84	0.357
**Occupation**						
Farmer/Agriculture	—	—		—	—	
Government worker	1.29	0.70, 2.37	0.407	1.23	0.62, 2.44	0.547
HCW-Government	2.31	0.91, 5.72	0.076	2.08	0.75, 5.59	0.154
Private Sector	1.98	1.23, 3.16	**0.005**	1.86	1.05, 3.28	**0.034**
HCW-private	0.99	0.28, 3.71	0.984	0.91	0.24, 3.63	0.890
Student	1.70	0.98, 2.93	0.057	1.67	0.87, 3.19	0.124
Trader/Self-employed	1.67	1.01, 2.78	**0.047**	1.51	0.84, 2.69	0.166
Unemployed	1.35	0.72, 2.51	0.346	1.17	0.59, 2.32	0.660
**Education**						
Bachelor	—	—		—	—	
Primary	0.78	0.36, 1.69	0.529	0.84	0.38, 1.86	0.671
High School	0.91	0.54, 1.55	0.738	0.94	0.54, 1.63	0.815
Junior High	1.04	0.73, 1.50	0.819	0.87	0.57, 1.33	0.516
Masters	0.93	0.51, 1.70	0.816	0.87	0.45, 1.66	0.669
No formal Edu	1.03	0.61, 1.74	0.912	0.90	0.50, 1.60	0.716
Vocational	0.57	0.15, 2.32	0.425	0.63	0.16, 2.68	0.526

Abbreviations: CI = Confidence Interval, OR = Odds Ratio, Significant *p*-values (*p* < 0.05) are shown in bold.

**Table 4:** Ordinal regression of antibiotic use practices and sociodemographic factors, Montserrado, Liberia, 2025 (N = 1160).

	Bivariate			Multivariate		
Characteristic	Crude OR	95% CI	*p*-Value	Adjusted OR	95% CI	*p*-Value
**Age group (years)**						
18–29	—	—		—	—	
30–39	0.99	0.75, 1.30	0.944	1.04	0.77, 1.42	0.777
40–49	1.26	0.92, 1.73	0.156	1.22	0.85, 1.74	0.278
50–59	1.03	0.70, 1.53	0.876	0.82	0.53, 1.28	0.391
60+	0.86	0.52, 1.43	0.558	0.60	0.33, 1.07	0.083
**Gender**						
Male	—	—		—	—	
Female	1.00	0.80, 1.26	0.983	1.06	0.82, 1.37	0.663
**Residence**						
Rural	—	—		—	—	
Urban	0.38	0.28, 0.52	**<0.001**	0.42	0.29, 0.61	**0.000**
**Occupation**						
Farmer/Agriculture	—	—		—	—	
Government worker	0.41	0.24, 0.68	**<** **0.001**	0.76	0.42, 1.36	0.355
HCW-Government	0.46	0.21, 1.02	0.056	1.00	0.42, 2.40	0.993
Private Sector	0.45	0.30, 0.67	**<0.001**	0.87	0.53, 1.41	0.564
HCW-private	0.67	0.22, 2.10	0.478	1.41	0.44, 4.65	0.564
Student	0.60	0.38, 0.96	**0.034**	1.12	0.64, 1.96	0.697
Trader/Self-employed	0.39	0.25, 0.61	**<0.001**	0.72	0.43, 1.18	0.191
Unemployed	0.54	0.32, 0.91	**0.022**	0.87	0.49, 1.57	0.650
**Education**						
Bachelor	—	—		—	—	
Primary	1.26	0.66, 2.45	0.491	1.04	0.53, 2.08	0.904
High School	0.65	0.42, 1.02	0.059	0.63	0.39, 1.02	0.059
Junior High	0.62	0.45, 0.83	**0.002**	0.67	0.47, 0.96	**0.029**
Masters	0.95	0.57, 1.59	0.834	0.86	0.50, 1.49	0.589
No formal Edu	0.56	0.36, 0.86	**0.009**	0.63	0.39, 1.02	0.063
Vocational	0.84	0.27, 2.71	0.768	0.79	0.25, 2.67	0.702

Abbreviations: CI = Confidence Interval, OR = Odds Ratio, Significant *p*-values (*p* < 0.05) are shown in bold.

## Data Availability

The data that support the findings of this study are available from the corresponding author, B.I.S., upon reasonable request.

## Supplementary Materials

The following supporting information can be downloaded at: https://www.mdpi.com/article/10.3390/antibiotics15070680/s1, Supplementary File S1: Study Questionnaire.

## Abbreviations

The following abbreviations are used in this manuscript:

ABR	Antibiotic resistance
AMR	Antimicrobial resistance
COM-B	Capability, Opportunity, Motivation–Behavior
HF	Health facility
KAP	Knowledge, attitudes, and practices
LMICs	Low- and middle-income countries
LRD	Liberian dollar
SD	Standard deviation
USD	United States dollar
WHO	World Health Organization
