# Supplementary material for: Knowledge, Attitudes, and Practices Related to Antibiotic Use and Antibiotic Resistance Among Adults in Communities of Montserrado County, Liberia: A Household-Based Cross-Sectional Study
Source: Antibiotics (Basel). 2026 Jul 10;15(7):680. doi: 10.3390/antibiotics15070680 (PMC13405560; doi:10.3390/antibiotics15070680)
Supplement: Supplementary file 1 [file antibiotics-15-00680-s001.zip › Supplementary File S1.pdf]

Evaluation of Antimicrobial Prescription, Antibiotic Susceptibility Pattern and Practices of Self-Medication in Montserrado County, North-Western Liberia

Interview with Household Member: Knowledge, Attitudes & Practices of Antibiotic Use Protocol

Household Code: ________________________ **Interview Date: __________________**

Area: ______________________________ **District: _____________________**

Community: _____________________

Section 1: Socio-Demographic

PID		Relation to the Head of Household	Age	Sex	
**Area of Residence:** Urban ☐ Rural ☐ **Health Insurance:** Yes ☐ No ☐					
**Education:** Illiterate ☐ Elementary ☐ Junior High School ☐ High School ☐ Bachelors ☐ Masters ☐ PhD ☐ Other ☐ ______________________________					
**01**	What is your occupation, that is, what kind of work do you mainly do?	Government worker ☐<br>Trader ☐<br>Private Sector Worker ☐<br>Farmer/Agriculture worker ☐<br>Healthcare worker ☐<br>Unemployed ☐<br>Student ☐<br>Housewife ☐			
**02**	What is your type of family?	Nuclear ☐<br>Extended ☐			
**03**	How many members are in your family (living in this household)?	Total……… Adults ___ Children ___<br>Male……………………………….<br>Female ……………………………			
**04**	Who is the decision maker in your family when it comes to health care?				
**05**	What is the average income of the family per year?				
**06**	How long does it take you from home to the nearest pharmacy (minutes/hours)?				

**07**	How long does it take you from home to the nearest healthcare facility (minutes/hours)?	

Section 2: Knowledge

Code	Questions	Coding Category
**08**	Have you ever heard of a type of medicine called antibiotics?	Yes ☐<br>No ☐
**09**	Yes (If yes, from where did you hear about them?	

Note: If the respondent says 'No" to question "09", please ask if they have heard of a widely used antibiotic in Liberia such as penicillin, ampicillin, erythromycin, ampiclox or metronidazole. If the response is yes, tick "Yes" in **Q 08**

Codes	Domains	Questions	Response				
			**SD**	**D**	**DK**	**A**	**SA**
**10**	Knowledge of the role of antibiotic	Antibiotics are useful for curing malaria					
		Antibiotics are useful for curing skin infections					
		Antibiotics are useful for curing ear infections					
		Antibiotics are useful for curing sore throat					
		Antibiotics are often needed for cold and flu illness					
		Diarrhea gets better faster with antibiotics					
**11**	Public knowledge about appropriate antibiotic use	Human body can become resistant to antibiotics after some time.					
		Antibiotics are effective against viral infections					
		Antibiotics increase the speed of recovery from colds					
		The utilization of antibiotics should last till disappear the symptoms regardless the time length					
		Antibiotics can distinguish between bacterial flora that normally live in the human body and pathogenic bacteria that cause diseases					

Codes	Domains	Questions	Response				
			SD	D	DK	A	SA
		Self-prescribed antibiotics do not have health consequences					
12	Side-effects of antibiotics	When wrongly/inappropriately used, antibiotics can kill good germs present in our bodies that help to protect us from infections					
		When antibiotics are wrongly/inappropriately used, they can lead to infections after killing good germs present in our bodies that help to protect us					
		Antibiotics can cause allergic reactions					
13	Antibiotic resistance	If bacteria cannot be killed by antibiotics, it can be very difficult to treat the infections they cause					
		Many infections nowadays become more difficult to treat as antibiotics are no longer as effective as they used to be					
		Misuse of antibiotics can lead to antibiotic resistance					
**SD – Strongly Disagree, D – Disagree, DK – Don't Know, A – Agree, SA – Strongly Agree**							

14. Have you ever previously taken antibiotics without a prescription? Yes ☐ No ☐ Don't know ☐

15. Can you mention the names of some OTC antibiotics that you often purchase?

____

____

16. If yes, what is the most common indication for which you take antibiotics without prescription?

Chest Cold ☐ Cough ☐ Common Cold/Running nose ☐ Diarrhea/Running stomach ☐

Skin rashes ☐ Ear pain/pus ☐ Sore Throat ☐ Fever ☐ Headache ☐

Urinary Tract Infection ☐

17. Did you take any antibiotics during 2023? Yes ☐ No ☐

18. If yes, what was your source of antibiotics? Prescribed by a physician ☐ Bought from a Pharmacy ☐ Leftover antibiotics previously bought and stored at home ☐ Got it from my friends ☐

Section 3: Attitudes

Codes	Domains	Questions	Response				
			SD	D	NS	A	SA
19	Preference for the use	When I have a cold, I should take antibiotics to prevent getting a more serious illness.					

Codes	Domains	Questions	Response				
			SD	D	NS	A	SA
	of antibiotics	When I get a fever, antibiotics help me to get better more quickly than paracetamol.					
		When I have pain in my throat, antibiotics help me to feel and get better.					
		I would take an antibiotic when I feel ill, even if I am not sure it is needed or recommended					
		I would not take an antibiotic when I feel ill and will wait and see if I get better without it					
**20**	Antibiotic resistance and safety	Whenever I take an antibiotic, I may contribute to the development of antibiotic resistance.					
		When I feel better, I discontinue taking antibiotics and do not complete the full course					
		Antibiotics have no harmful effects on the body, hence they can be used frequently.					
		I only take antibiotics for fever, cold, etc. if it is prescribed by a doctor					
**21**	Attitudes to doctor's prescribing of antibiotics	I expect to receive an antibiotic whenever I visit a doctor.					
		For whatever reason, I am less satisfied with a doctor's visit if I do not receive an antibiotic.					
		If a doctor does not prescribe an antibiotic when I think one is needed, I will go to another doctor.					
**22**		I suggest to the doctor to include antibiotics in my prescription if he doesn't do so on his/her own					
**23**	If yes, to the above question then, why? What do you think will be the benefit of it?						
**SD – Strongly Disagree, D – Disagree, NS – Not Sure, A – Agree, SA – Strongly Agree**							

Section 4: Practices

Codes	Questions	Response				
		A	MT	S	R	N
**24**	If you feel better after taking a few doses of antibiotics, will you still complete the full course of the prescribed antibiotics?					
**25**	If you have an illness such as sore throat, fever, cold cough, or running nose, do you prefer to take antibiotics?					
**26**	When you have an illness such as sore throat, fever, cold cough, or running nose and desire to take an antibiotic, do you prefer to obtain it from the pharmacy rather than going to a doctor/healthcare worker?					

Codes	Questions	Response				
		A	MT	S	R	N
27	Do you consult a doctor before starting an antibiotic?					
28	Do you follow the pharmacist/drug store attendant instructions on how to take antibiotics when using it?					
29	Do you check the expiry date of the antibiotic before using it?					
30	Do you use antibiotics to prevent you from getting ill?					
31	Do you inform the doctor that you have previously taken an antibiotic for the same illness before your visit to the doctor?					
**A – Always, MT – Most Times, S – Sometimes, R – Rarely, N – Never**						

32. In general, where do you usually obtain information about utilizing antibiotics?

Physician ☐ Pharmacist ☐ Leaflet ☐ Previous experience ☐ Friends ☐

33. Usually, how long do you take antibiotics? 1-3 days ☐ 4-7 days ☐ >7 days ☐ until symptoms disappear ☐

34. If you often use antibiotics, can you mention the names of some that you often use? ____________

____

35. Do you keep antibiotics as reserve medicine at home (Ask them to show their reserve medicine box or whatever it is and note down the available medicines available in their homes)? Yes ☐ No ☐

Details of antibiotics present in the reserve.

Brand name	Generic name	No. of units available	Prescription Present	Date of prescription	Expiry date	For whom were they bought	For what purpose

36. Storage condition (stored in packs separately or mixed with other medicines, temperature –cool or moist):
____
____
